# Mining significant high utility gene regulation sequential patterns

**DOI:** 10.1186/s12918-017-0475-4

**Published:** 2017-12-14

**Authors:** Morteza Zihayat, Heidar Davoudi, Aijun An

**Affiliations:** 1 Ted Rogers School of Information Technology Management, Ryerson University, Bay Street, Toronto, Canada; 2 Department of Electrical Engineering and Computer Science, York University, Keele Street, Toronto, Canada

**Keywords:** High utility pattern mining, Gene regulation sequential patterns, Time-course microarray datasets

## Abstract

**Background:**

Mining frequent gene regulation sequential patterns in time course microarray datasets is an important mining task in bioinformatics. Although finding such patterns are of paramount important for studying a disease, most existing work do not consider gene-disease association during gene regulation sequential pattern discovery. Moreover, they consider more absent/existence effects of genes during the mining process than taking the degrees of genes expression into account. Consequently, such techniques discover too many patterns which may not represent important information to biologists to investigate the relationships between the disease and underlying reasons hidden in gene regulation sequences.

**Results:**

We propose a utility model by considering both the *gene-disease association* score and their *degrees of expression levels* under a biological investigation. We propose an efficient method called *Top-HUGS*, for discoverying *significant high utility gene regulation sequential patterns* from a time-course microarray dataset.

**Conclusions:**

In this study, the proposed methods were evaluated on a publicly available time course microarray dataset. The experimental results show higher accuracies compared to the baseline methods. Our proposed methods found that several new gene regulation sequential patterns involved in such patterns were useful for biologists and provided further insights into the mechanisms underpinning biological processes. To effectively work with the proposed method, a web interface is developed to our system using Java. To the best of our knowledge, this is the first demonstration for *significant high utility gene regulation sequential pattern discovery*.

## Background

Microarrays have been extensively used for discovering differentially expressed genes in human diseases. Several methods have been designed to observe massive gene expressions and recognize their regulations during a clinical study. However, several studies show that a disease cannot be characterized by a single gene but emerges as complex interactions among multiple genetic variants [1]. Gene regulation sequential pattern analysis is an important task for studying illness events such as cancer formation. The formation of such diseases happen over a time period, hence abnormal alternations can be identified by monitoring gene expressions over a period of time. Although associations among these genes are important, existing work only consider how to discover differentially expressed genes over time. Proposed by several studies [1–4], *sequential pattern mining* is an effective technique to discover such associations as gene regulation sequential patterns.

Given a dataset, where each record is a sequence containing a list of items/itemsets, *Sequential pattern mining* is the process of discovering sequences of items/itemsets whose occurrence in the dataset is no less than some pre-defined threshold. Such techniques can identify a potential gene regulation sequential pattern if it occurs frequently (i.e., more than the threshold) in a period of time.

Although sequential pattern mining has been applied to identify gene regulation sequential patterns, several shortcomings can be found in the existing algorithms. First, the techniques usually select significant sequences based on the *frequency/support* framework. Hence, those patterns whose frequency is relatively high, are considered as significant patterns. Nonetheless, as clinical studies have indicated, the frequency by itself might not be adequately informative to find significant sequences with respect to a certain disease. For instance, several genes tend to be more substantial compared to others in causing a specific disease and some genes are certainly more effective compared to others in fighting a disease. Furthermore, majority of the existing techniques consider the more general up/down effects of gene’s behavior (i.e., gene expression) in a microarray dataset by transforming the expression value to *highly expressed* or *highly repressed* and do not consider the level of expressions. For example, a gene might not appear frequently but its behavior is extremely remarkable in each appearance or vice versa. Consequently, sequences that have highly expressed/repressed genes might not be identified by the frequency based methods since such approaches neither take into account the significance of genes, nor the degrees of expression under a biological investigation.

To address these limitations, the *utility* is introduced to sequential pattern mining. Utility is a domain driven function that can be defined based on an objective. *High utility sequential pattern (HUSP)* mining is the process of discovering sequential patterns with respect to the utility function. In this context, a sequence is a *high utility sequential pattern*, if its utility in a dataset is no less than a *minimum utility threshold*. Although such approaches can be helpful to discover significant gene regulation sequential patterns, most existing *HUSP* mining methods are mainly studied to discover patterns in market basket analysis (e.g., finding profitable customer shopping behavior), and have not been deployed to discover patterns from complex sequential datasets such as time course microarray datasets. The main challenge to apply this approach is i) how to model the utility such that it represents the objective (e.g., a specific disease) effectively and ii) how to convert input sequential dataset (e.g., a time course microarray dataset) to a utility-based sequential database. Second, in gene regulation sequential pattern discovery, setting an effective value for the threshold is not easy for biologists. If the threshold is set too low, a large number of patterns can be discovered, which makes it hard to analyze the mined patterns. On the other hand, if the threshold is set too high, some interesting patterns may be missed. A practical solution to find significant patterns is to set a bound on the size of output (e.g., top-k patterns). Given k as the size of output, the algorithm should basically search for patterns with a very low threshold (e.g., zero or a value close to zero) to guarantee that at least *k* patterns can be found. Finding patterns with a very low threshold causes very high computational costs. The main challenge is how to raise the threshold quickly while no top-k pattern is missed.

In this paper, we address the aforementioned issues by introducing a utility model that considers the *gene importance* and its *degrees of expression* under a biological investigation. To the best of our knowledge, our proposed method in [5] is the only work to discover *utility-based gene regulation sequential patterns* in a time course microarray dataset. In this paper, we design an algorithm called *Top-HUGS* to mine top-k high utility gene regulation sequential patterns (to be defined later) by considering the proposed utility model. *Top-HUGS* takes *k* (i.e., number of output patterns) and a disease (as the objective) as input and it finds the top *k* most important gene regulation sequential patterns from a time course microarray dataset. We prove that *Top-HUGS* does not miss any top-k high utility gene regulation sequences, which take place across different time points during the course of biological observations. Our contributions are summarized as follows.

- We define a utility model based on the *importance of genes* with respect to a disease and their *finer degrees of expression* under a biological investigation.
- We present the problem of *threshold free high utility gene regulation sequential pattern mining*. We design several adjusting strategies to initialize and raise the threshold before and during the mining process. We prove that the proposed strategies do not miss true high utility patterns.
- We propose a new algorithm called *Top-HUGS* to mine top-k high utility gene regulation sequences from a time course microarray dataset without any given threshold.
- We conduct experiments on a real and publicly available time course microarray dataset to evaluate the effectiveness and efficiency of *Top-HUGS* to find the patterns with respect to three different diseases.
- A web interfece is designed and implemented for our proposed algorithms. This is the first demonstration for mining high utility gene regulation sequential patterns. Demo available at http://mzk.eecs.yorku.ca:8080/GeneAssociation/

The rest of the paper is organized as follows. We present a summary of related work. Then, the proposed method is discussed. We evaluate the proposed methods and finally we conclude the paper.

## Related work

In this section, we describe some existing work on sequential pattern mining, high utility sequential pattern mining, and sequential pattern mining in Bioinformatics.

### Sequential pattern mining

Mining *sequential patterns* in sequence databases is a challenging problem in data mining [6–11], which was first introduced by Agrawal et al. [7]. A *subsequence* is called *sequential pattern* or *frequent sequence* if it frequently appears in a sequence database, and its frequency is no less than a user-specified *minimum support threshold* [7]. In the last two decades, several algorithms have been proposed such as *AprioriAll* [6], *GSP* [10], *FreeSpan* [8], *PrefixSpan* [9], *SPADE* [11] and *SPAM* [7]. These algorithms can be generally categorized as using a horizontal database (e.g., *AprioriAll*, *GSP*, *FreeSpan* and *PrefixSpan*) or a vertical database (e.g., *SPADE* and *SPAM*). A vertical representation provides the advantage of calculating frequencies of patterns without performing costly database scans. This allows vertical mining algorithms to perform better on dense dabases or long sequences than algorithms using the horizontal format. The *AprioriAll* and *GSP* algorithms use candidate-generation-and-test methodology for mining sequential patterns. *FreeSpan* and *PrefixSpan* discover sequential patterns by the pattern-growth methodology. The *SPADE* and *SPAM* algorithms use different vertical representations for mining sequential patterns.

### High utility sequential pattern mining

Although sequential pattern mining algorithms have been applied to solve many real-world problems [12], they treat all items as having the same importance and assume that an item appears at most once at any time point, which is not the case for many applications. Recently, High Utility Pattern (HUP) mining was proposed to address such limitations to patterns (itemsets or sequences) whose utility is no less than a minimum utility threshold.

High utility pattern mining [13–17] considers the external utility (e.g., unit profits) and internal utility (e.g., quantity) of items such that it provides users with patterns having a high utility (e.g., profit). Some efficient algorithms such as *two-phase* [18], *IHUP* [19], *UP-Growth* [20], *HUI-Miner* [21] and *FHM* [22] have been proposed to find high utility itemsets (*HUIs*) from a transaction database, where the sequential ordering of itemsets is not considered. The addition of ordering information makes the pattern mining problem fundamentally different and much more challenging than mining high utility itemsets. The concept of *high utility sequential pattern (HUSP)* mining was first introduced by Ahmed et al. [13], who defined an over-estimated sequence utility measure, *SWU* (i.e., Sequence-Weighted Utility), which has the downward closure property, and proposed two approaches, called *UL* and *US*, to find *HUSPs* based on *SWU*. *UL* is a level-wise candidate generation-and-testing algorithm and hence involves multiple scans of the database and generates a large number of *high-SWU* candidate sequences. They also proposed *US* which uses a pattern-growth method inspired by *PrefixSpan* [9] to generate all sequences whose *SWU* satisfies the threshold, and then scans the database again to compute the exact utilities of *high-SWU* candidate sequences to find *HUSPs*. Shie et al. [15] proposed a framework for mining *HUSPs* in a mobile environment. Their algorithm can only handle sequences with a single item in each sequence element. Ahmed et al. proposed efficient algorithms for mining *high utility access sequences* from web log data [14], which also only consider single-item sequences. Recently, Yin et al. [16] proposed the *USpan* algorithm for mining *HUSPs*. They used a lexicographic tree to extract the complete set of high utility itemset-sequences and designed mechanisms for expanding the tree with two pruning strategies. One of the pruning strategy is based on *SWU*. The other pruning strategy needs to be used after candidate generation. Moreover, it needs to construct *Utility Matrix* (the proposed data structure) for each generated sequence and also it traverses each element once to calculate the utility of extended sequence, which is very time consuming.

### Sequential pattern mining in Bioinformatics

Sequential pattern mining has been widely applied to the bioinformatics domain for finding patterns of certain elements in genes, for predicting protein function, for analyzing gene expression, for motif discovery in DNA sequences and for discovering sets of genes that are frequently co-occurred in most biological conditions in a microarray dataset. Some of these methods are *apriori algorithm* [23], *half-spaces* [4], and *FPtree algorithm* [24]. Moreover, in [25], a method, called *MAGIIC*, is proposed to discover the structure motifs from protein sequences. In [2], the authors propose an algorithm called *CTGR-Span (Cross-Timepoint Gene Regulation Sequential pattern)* to efficiently discover *CTGR-SPs (Cross-Timepoint Gene Regulation Sequential Patterns)*. However, to the best of our knowledge, all of the aforementioned methods do not consider the objective of the study. That is, the temporal behavior of genes under a biological investigation is ignored in the problem setting, so is the importance of genes with respect to a disease.

Several work have been also studied the relationships between genes and a specific disease [1]. However, such methods ignore the sequential relationships among genes and only study the behavior of each gene individually. In [3], the authors design an algorithm to identify novelty in sequential patterns with respect to a disease (e.g., Alzheimer). However, they ignore time course sequential databases and also the proposed method still discovers pattern based on frequency.

To the best of our knowledge, our proposed method in [5] is the only work to learn *utility-based gene regulation sequential patterns* in a time course microarray dataset. In this paper, we extend [5] as follows. First, we improve *TU-SEQ* by proposing a new strategy to raise the threshold which results a new method called *Top-HUGS* to find top-k high utility gene regulation sequential patterns efficiently. The correctness of the proposed method is proved. Second, the newly proposed algorithm is compared with the algorithm in [5] in the experiments. Third, the experimental results are extended by (1) adding the results of two more disease (i.e., *Asthma* and *Rheumatoid Arthritis*) and (2) evaluating the effectiveness of the newly proposed strategy.

## Methods

In this section, we present how to find high utility gene sequential patterns from a time course microarray dataset. The process consists of three main parts: 1) We transform a microarray dataset to a sequential database, 2) We define and present the problem of top-k high utility gene regulation sequential pattern mining, and 3) We design algorithms to solve the problem.

### Converting a time course microarray dataset to a time course sequential dataset

We first propose a procedure to transform a time course microarray dataset to a proper *time course sequential dataset*.

Table 1 presents a time course microarray dataset obtained from three patients whose IDs are *P*_1_, *P*_2_ and *P*_3_. As Table 1 shows, each row represents values of three genes *G*_1_, *G*_2_ and *G*_3_ over four time point samples. *T* *S*_1_, *T* *S*_2_, *T* *S*_3_ and *T* *S*_4_.

**Table 1:** An example of a time course microarray dataset

Patient IDs	Genes	*T* *S*_1_	*T* *S*_2_	*T* *S*_3_	*T* *S*_4_
*P*_1_	*G*_1_	2420	546	100	50
	*G*_2_	321	98	454	974
	*G*_3_	410	350	251	243
*P*_2_	*G*_1_	128	786	135	344
	*G*_2_	253	820	482	90
	*G*_3_	290	150	256	864
*P*_3_	*G*_1_	600	188	99	40
	*G*_2_	500	555	510	80
	*G*_3_	200	400	350	450

The real values expressed for a gene in each time sample can present a gene’s *temporal behavior*. In order to derive the temporal behavior of each gene at each time sample, we take the first time sample as a baseline. As such, the *temporal behavior* of a gene at a time sample *TS* equals to the expression value of the gene at *TS* divided by the expression value of the gene at the first time sample. This value shows the degree of expression of the gene at time sample *TS*. Table 2 shows the temporal behavior values as a fold change matrix.

**Table 2:** Fold changes of gene/probe values

Patient IDs	Genes	*T* *S*_1_	*T* *S*_2_	*T* *S*_3_	*T* *S*_4_
*P*_1_	*G*_1_	1	2.2	-2.4	-4.8
	*G*_2_	1	-3.2	1.4	3.0
	*G*_3_	1	-1.1	-1.6	-1.6
*P*_2_	*G*_1_	1	6.1	1.0	2.6
	*G*_2_	1	3.2	1.9	-2.8
	*G*_3_	1	-1.9	-1.1	2.9
*P*_3_	*G*_1_	1	-3.1	-6.6	-15
	*G*_2_	1	1.1	1.0	-6.2
	*G*_3_	1	2	1.7	2.2

Given Table 2 and a threshold *γ*, we convert each expression value as *up-regulated* (showing by + meaning that the value is greater than *γ*), *down-regulated* (showing by − meaning that the value is less than - *γ*), or *normal* (neither *up-regulated* nor *down-regulated*). Then, we preserve the gene expressions that are either up-regulated or down-regulated. Note that, this threshold is useful to filer out noisy behavior. Each gene (i.e., *G*_*x*_) in a sample can be thought of as being one of two *items*, one item referring to the gene being up (i.e., *G*_*x*+_), the other referring to the gene being down (i.e., *G*_*x*−_).

Given *γ*=1.5, Table 3 represents the transformed dataset (i.e., the *time-course sequential* dataset). In this table, given patient *P*_1_, up-regulated *G*_1+_(2.2) and down-regulated *G*_2−_(3.2) which occurred at the time *T* *S*_2_ and their temporal behavior values (as defined above) are 2.2 and 3.2 respectively.

**Table 3:** A time course sequential dataset from time course microarray dataset in Table 1

Patiend IDs	Sequence
*P*_1_	$\{G_{1^{+}}(2.2)G_{2^{-}}(3.2)G_{3^{-}}(1.1)\}_{2} \{G_{1^{-}}(2.4)G_{2^{+}}(1.4)G_{3^{-}}(1.6)\}_{3} \{G_{1^{-}}(4.8)G_{2^{+}}(3.0)G_{3^{-}}(1.6)\}_{4}$
*P*_2_	$\{ G_{1^{+}}(6.1) G_{2^{+}}(3.2)G_{3^{-}}(1.9)\}_{2} \{G_{1^{+}}(1.0)G_{2^{+}}(1.9)G_{3^{-}}(1.1)\}_{3} \{G_{1^{+}}(2.6)G_{2^{-}}(2.8)G_{3^{+}}(2.9) \}_{4}$
*P*_3_	$\{G_{1^{-}}(3.1) G_{2^{+}}(1.1) G_{3^{+}}(2.0)\}_{2} \{G_{1^{-}}(6.6) G_{2^{+}}(1.0) G_{3^{+}}(1.7)\}_{3} \{G_{1^{-}}(15) G_{2^{-}}(6.2) G_{3^{+}}(2.2) \}_{4}$

### Problem statement

Let *G*={*G*_1+_,*G*_1−_,*G*_2+_,*G*_2−_,...,*G*_*n*+_,*G*_*n*−_} be a set of distinct gene regulation items. A *geneset* *GS* is a set of gene regulation items. A *time-course sequential* dataset consists of patients {*P*_1_,*P*_2_,....,*P*_*K*_}, where each patient has an identifier *P*_*r*_ and is represented as an ordered list of *time point samples (or in brief time samples (TSs))*. Each time sample is a geneset. We denote the time sample *T* *S*_*d*_ of *P*_*r*_ as $P_{r}^{d}$.

#### **Definition 1**

The **importance of gene** ***g*** is computed based on one or more disease-dependent variables *v* *a* *r*_1_,*v* *a* *r*_2_,...,*v* *a* *r*_*k*_. Therefore, *Gene Importance (GI)* is defined as *G* *I*(*g*)=*f*_*g*_(*v* *a* *r*_1_,*v* *a* *r*_2_,...,*v* *a* *r*_*k*_), where *f*_*g*_ is the function for calculating the importance of *g*.

Table 4 illustrates the genes importance with respect to a disease. For simplicity and without loss of generality, we assume that the importance of *G*_*x*_ represents the importance of both *G*_*x*+_ and *G*_*x*−_.

**Table 4:** Importance of genes

Gene	*G*_1_	*G*_2_	*G*31
Score	0.8	0.6	0.1

#### **Definition 2**

**Internal utility** is a *temporal behavior* of a gene *g* in the time sample *T* *S*_*d*_ of patient $P_{r}\left (i.e., P_{r}^{d}\right)$. It is denoted as $IGU_{dis}\left (g,P_{r}^{d}\right)$ and is defined as the expression value of *g* at *T* *S*_*d*_ divided by the expression value of *g* at the first time sample in *P*_*r*_.

For example, in Table 3, given gene *G*_1−_ and time sample *T* *S*_3_ in sequence *P*_1_, $IGU\left (G_{1^{-}}, P_{1}^{3}\right) = 2.4$. This value specifies the relative abundance of the gene in the time sample.

#### **Definition 3**

Given disease *dis*, the **utility of gene** ***g*** **in time sample** $P_{r}^{d}$ is defined as: $GU\left (g,P_{r}^{d}\right) = f_{gu}\left (GI(g),IGU\left (g,P_{r}^{d}\right)\right)$, where *f*_*gu*_ is the function to compute the utility.

For simplicity, we assume that *f*_*gu*_ is calculated as $f_{gu}\left (GI(g),IGU\left (g,P_{r}^{d}\right)\right) = GI(g)\cdot IGU\left (g,P_{r}^{d}\right)$.

#### **Definition 4**

The **utility of a geneset** ***GS*** **in a time sample** ***T*** ***S***_***d***_ **of a patient** ***P***_***r***_ where *G* *S*⊆*T* *S*_*d*_, is defined as $GU\left (GS, P_{r}^{d}\right) = \sum \limits _{g \in GS}GU\left (g, P_{r}^{d}\right)$.

#### **Definition 5**

(**Occurrence of a sequence** ***α*** **in a patient** ***P***_***r***_ **)** Given a patient $P_{r} = \langle P_{r}^{1}, P_{r}^{2},..., P_{r}^{n}\rangle $ and a gene regulation sequence *α*=〈*G* *S*_1_,*G* *S*_2_,...,*G* *S*_*Z*_〉 where $P_{r}^{i}$ is a time sample and *G* *S*_*i*_ is a geneset, *α* occurs in *P*_*r*_ iff there exist integers 1≤*e*_1_<*e*_2_<...<*e*_*Z*_≤*n* such that $GS_{1}\subseteq P_{r}^{e_{1}}, GS_{2} \subseteq P_{r}^{e_{2} },..., GS_{Z} \subseteq P_{r}^{e_{Z}}$. The ordered list of genesets $\langle P_{r}^{e_{1}},P_{r}^{e_{2}},..., P_{r}^{e_{Z}}\rangle $ is called an *occurrence of α in P*_r_. The set of all occurrences of *α* in *P*_*r*_ is represented as *O* *c* *c* *S* *e* *t*(*α*,*P*_*r*_).

#### **Definition 6**

(The **utility of a gene regulation sequential pattern** ***α*** **in a patient sequence** ***P***_***r***_ **)** Let $\tilde {o} = \langle P_{r}^{e_{1}}, P_{r}^{e_{2}},..., P_{r}^{e_{Z}}\rangle $ be an occurrence of *α*=〈*G* *S*_1_,*G* *S*_2_,...,*G* *S*_*Z*_〉 in the sequence *P*_*r*_. The utility of *α* w.r.t. $\tilde {o}$ is defined as $GU(\alpha,\tilde {o}) = \sum \limits _{i=1}^{Z} GU\left (GS_{i},P_{r}^{e_{i}}\right)$. The utility of *α* in *P*_*r*_ is defined as $GU(\alpha, P_{r}) = \max \{GU(\alpha, \tilde {o})\mid \tilde {o} \in OccSet(\alpha, P_{r})\}$.

#### **Definition 7**

The **(utility of a gene regulation sequence** ***α*** **in a time course sequential dataset** ***D*** **)** The utility of a gene regulation sequence *α* in a time course sequential dataset *D* is defined as $GU(\alpha, D) = \sum \limits _{P_{r} \in D} GU(\alpha, P_{r})$.

#### **Definition 8**

**(High Utility Gene regulation Sequence (HUGS))** Given a threshold *δ*, a sequence *α* is a *High Utility Gene Regulation Sequence* (HUGS) in a time course sequential dataset *D*, iff *G* *U*(*α*,*D*) is no less than *δ*.

#### **Definition 9**

**(Top-k High Utility Gene regulation Sequence in a time course sequential dataset** ***D*** **)** A gene regulation sequence *α* is called a top-k High Utility Gene Regulation Sequence (*HUGS*) in *D*, if there are less than *k* sequences whose utility value in *D* is no less than *G* *U*(*α*,*D*).

**Problem Statement.** Given a time course sequential dataset *D* and *k* as the number of output patterns, the problem of finding the threshold free high utility gene regulation sequential patterns is to identify all the patterns whose utility is no less than *m* *i* *n* *U* *t* *i* *l*_*opt*_, where *m* *i* *n* *U* *t* *i* *l*_*opt*_= min{*G* *U*(*β*,*D*)|*β*∈*T* *H* *U* *G* *S*_*D*_} and *T* *H* *U* *G* *S*_*D*_ is the set of top-k HUGSs over *D*.

## Mining top-k high utility gene regulation sequential patterns

In this section, we present our proposed algorithm for mining top-k high utility gene regulation sequential patterns. The propose algorithm is an extended version of our proposed algorithm in [5], called *TU-SEQ (Top-k Utility-based gene regulation SEQuential pattern discovery)*. We first present an overview of *TU-SEQ*. Then we propose the extended version of TU-SEQ, called *Top-HUGS*, by proposing a novel strategy to raise the threshold during the mining process.

### An overview of *TU-SEQ*

*TU-SEQ* takes *k* as an input parameter and returns top-*k* sequences with the highest utility in a time course sequential dataset *D*. It uses two main data structures called *ItemUtilLists* and *HUSP-Tree* to preserve the information of potential top-k HUGSs.

Below, we describe *ItmeUtilLists* and *HUSP-Tree*. For more details about the data structures, readers can refer to [5].

**Fig. 1:**
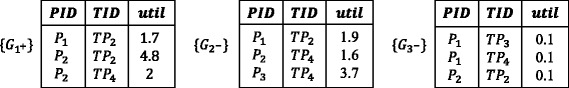
ItemUtilLists of *G*_1+_, *G*_2−_ and *G*_3−_ in Tables 3 and 4

The *ItemUtilLists* of a gene *G* has several rows. Each row keeps the utility information of gene *G* in the time sample $P_{v}^{u}$ that contains *G*. Each row consists of three fields: *PID*, *TID* and *util*. *PID* and *TID* store the identifiers of *P*_*v*_ and *T* *S*_*u*_, respectively and *util* stores the utility of *G* in $P_{v}^{u}$ (Definition 3). Figure 1 illustrates the *ItemUtilLists* of *G*_1+_, *G*_2−_ and *G*_3−_ in Table 3.

**Fig. 2:**
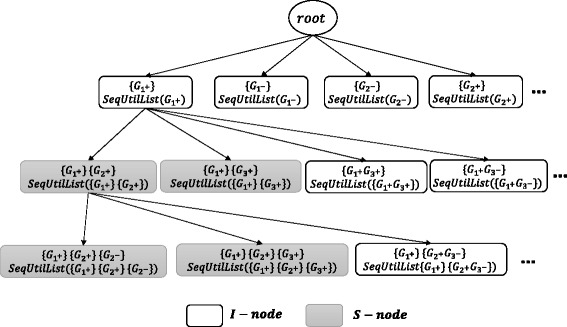
An example of HUSP-Tree for the dataset in Tables 3 and 4

A **HUSP-Tree** is a tree data structure where each non-root node shows a sequence of genesets. Figure 2 shows part of the *HUSP-Tree* for the the dataset in Table 3, where the root is empty. *HUSP-Tree* is a lexicographic tree. That is, a node at the first level represents a sequence of length 1, a node at the second level represents a 2-sequence, and so on. We design a non-root node to maintain information about the sequence in a field called *SeqUtilList*. The *sequence utility list (SeqUtilList)* of a sequence *α* is a list of three-value tuples 〈*P* *I* *D*,*T* *I* *D*,*u* *t* *i* *l*〉 represents an occurrence of *α* in a sequence of the dataset and the utility of *α* with respect to the occurrence. The *PID* in the patient ID in which *α* occurs, *TID* is the ID of the last time sample in the occurrence of *α*, and *util* is the utility value of *α* with respect to the occurrence. The *SeqUtilList* of *α* is denoted as *S* *e* *q* *U* *t* *i* *l* *L* *i* *s* *t*(*α*).

There are two types of non-root node in a *HUSP-Tree*: *I-node* or *S-node*. The nodes are constructed using two main steps *I-Step* and *S-Step*, which add *I-nodes* and *S-nodes* to the tree respectively.

#### **Definition 10**

**(I-Step)** Given a sequence pattern *α*, I-Step adds a gene *G* into the last geneset of *α* (denoted as *α*⊕*G*). The node with the output pattern is called *I-node*.

#### **Definition 11**

**(S-Step)** Given a sequence *α*, S-Step generates a new pattern by adding a geneset {*G*} after the last geneset of *α* (denoted as *α*⊗*G*). The node with the output pattern node is called *S-node*.

In Fig. 2, given *α*={*G*_1+_}, the node for sequence {*G*_1+_ *G*_3+_} is an *I-node*, while the node for {*G*_1+_}{*G*_3+_} is a *S-node*.

For more details about the tree construction, readers can refer to [5].

*TU-SEQ* uses a structure called *TKList* to store the information of top-k high utility gene regulation sequential patterns.

#### **Definition 12**

**Top-k HUGS List (TKList**) is a fixed-size sorted list which maintains the top-k high utility gene regulation sequential patterns and their *utility* values. Each tuple in *TKList* has two elements: 〈*α*,*u* *t* *i* *l*〉, where *α* is the pattern and *util* is the utility of pattern *α* in the dataset.

Since *TU-SEQ* is a threshold free mining approach, the threshold is not given as an input parameter. Hence, *TU-SEQ* uses a variable called *minUtil* to keep track of the current threshold which is initially set to zero. *TU-SEQ* uses *minUtil* to remove candidates which are not top-k HUGS.

*TU-SEQ* is a combination of a baseline procedure and one raising threshold strategy. Below, we first present the baseline procedure.

Given a time course sequential dataset *D* and *k*, TU-SEQ first sets *minUtil* to 0. Then, it builds *ItemUtilList* and *HUSP-Tree* by applying the *S-Step* and *I-Step* procedures. Once a new node is inserted to *HUSP-Tree*, the pattern preserved by the node and its *utility* are inserted as a new tuple to *TKList*. It keeps inserting more patterns till *k* valid patterns are inserted. Then, the *minUtil* is increased to the *util* value of the pattern with the lowest *util* in *TKList*. The updated *minUtil* is used to remove the search space when searching for more patterns. Thereafter, once a new node is added to the tree, *TKList* is updated. Accordingly, the patterns with *util* less than *minUtil* are removed from *TKList*. Given the updated *TKList*, *minUtil* is updated according to the the *util* value of the *kth* pattern in the list. This procedure continues until no more nodes are added to the tree. That is, the top-k *HUGSs* are found in the dataset.

While the proposed baseline procedure can find the top-k high utility gene regulation sequential patterns correctly, it is not efficient approach since it generates to many candidates. The main shortcoming is that *minUtil* starts from 0. TU-SEQ addresses this problem by using an effective strategy, called *PES (Pre-Evaluation using 1-sequences and sequences) strategy*, for initializing the threshold before *HUSP-Tree* construction to improve the performance.

*PES* initializes *TKList* by adding the *utility* of genes and sequences in the dataset to the *TKList* before the tree construction. In [5], we present theoretical aspects of this strategy. Once all the items in the dataset *D* are added to *ItemUtilList*, *PES* computes the *utility* of each gene and each sequence. Given the updated *TKList*, TU-SEQ initializes *minUtil* by the *util* value of kth tuple in *TKList*.

#### **Example 1.**

Given *k*=4, the time course sequential dataset *D* in Table 3, the *utility* of gene *G*_1+_ in *D* is computed as follows: *G* *U*(*G*_1+_,*D*)=*G* *U*(*G*_1+_,*P*_1_)+*G* *U*(*G*_1+_,*P*_2_)+*G* *U*(*G*_1+_,*P*_3_)=1.76+4.88+0=6.64. We can calculate the *utility* of the other genes similarly, *G* *U*(*G*_1−_,*D*)=15.84, *G* *U*(*G*_2+_,*D*)=3.72, *G* *U*(*G*_2−_,*D*)=7.32, *G* *U*(*G*_3+_,*D*)=0.51 and *G* *U*(*G*_3−_,*D*)=0.35. Moreover, the *utility* of each sequence is calculated using *ItemUtilLists*. Once *D* is scanned, *P*_1_ and its *utility* (e.g., 11.5) are added to the *TKList* as the other sequences in *D* (e.g., *P*_2_ (12.18) and *P*_3_(25.07)). Given the three sequences, six genes and their *utility* values, the *util* values in the *TKList* are {25.7,15.84,12.18,11.5}. Therefore, *m* *i* *n* *U* *t* *i* *l*=11.5 after applying *PES* strategy.

Applying *PES* strategy TU-SEQ effectively raises the minimum threshold to a reasonable level before the tree construction, and prevents generating unpromising candidates.

## Top-HUGS: top-K high utility gene regulation sequential pattern mining

In this section, we improve TU-SEQ by proposing a new algorithm, called Top-HUGS, which applies another raising threshold strategy called *RSO* (Raising threshold by Sorting concatenation Order) to effectively reduce computational overhead and efficiently raise the threshold. As mentioned, during the mining process we apply two concatenation processes to create candidates: *I-Concatenate* and *S-Concatenate*. In the threshold-based method, the processes are used in a depth-first manner. For example, in Fig. 2, we start from *root*. Then, one of the searching paths is $\langle \rangle \rightarrow \langle G_{1^{+}} \rangle \rightarrow \langle G_{1^{+}}G_{2^{+}} \rangle \rightarrow \langle \{G_{1^{+}}\}\{G_{2^{+}}G_{3^{-}}\}\rangle \rightarrow \dots \} $. Once this path is over, it goes for other branches until more new patterns left. Since the threshold is given, any order of generating candidates will result the same number of candidates. However, in top-k methods, the order of concatenating items does matter. This is due to the fact that, we are raising the threshold based on the utility of the patterns added to the *TKList*. Therefore, early finding of the candidates with higher utility during the mining process can raise the threshold sooner and as a result less candidates will be generated.

Before we present how RSO works, we first present a few definitions [26].

### **Definition 13**

(**First occurrences of a sequence** ***α*** **in a patient** ***P***_***r***_ **)** Given a patient $P_{r} = \langle P_{r}^{1}, P_{r}^{2},..., P_{r}^{n}\rangle $ and a sequence *α*=〈*G* *S*_1_,*G* *S*_2_,...,*G* *S*_*Z*_〉, $\tilde {o}\in OccSet(\alpha,P_{r})$ is the first occurrence of *α* in *P*_*r*_, iff the last geneset in $\tilde {o}$ occurs sooner than the last geneset of any other occurrence in *O* *c* *c* *S* *e* *t*(*α*,*P*_*r*_).

### **Definition 14**

**(Rest sequence of patient** ***P***_***r***_ **w.r.t. sequence** ***α*** **)** Given a patient $P_{r} = \langle P_{r}^{1}, P_{r}^{2},..., P_{r}^{n}\rangle $ and *α*=〈*G* *S*_1_,*G* *S*_2_,...,*G* *S*_*Z*_〉, where *α*≼*P*_*r*_. The rest sequence of *P*_*r*_ w.r.t. *α*, is defined as: $restSeq(P_{r},\alpha) = \langle P_{r}^{m}, P_{r}^{m+1},..., P_{r}^{n}\rangle $, where $P_{r}^{m}$ is the last geneset of the first occurrences of *α* in *P*_*r*_.

### **Definition 15**

**(Upper utility of a sequence** ***α*** **in a patient** ***P***_***r***_ **)** The rest utility of *α* in *P*_*r*_ is defined as *Ψ*(*α*,*P*_*r*_)=*G* *U*(*α*,*P*_*r*_)+*G* *U*(*r* *e* *s* *t* *S* *e* *q*(*P*_*r*_,*α*)).

For example, given *α*=〈{*G*_1+_ *G*_2−_}{*G*_1−_}〉 and *P*_1_ in Table 3, *r* *e* *s* *t* *S* *e* *q*(*P*_1_,*α*)=〈{〈{*G*_1−_(2.4)*G*_3−_(1.6)}{*G*_1−_(4.8)*G*_2+_(3.0)*G*_3−_(1.6)}〉}〉. Hence, *G* *U*(*r* *e* *s* *t* *S* *e* *q*(*P*_1_,*α*))=6.68, then *Ψ*(*α*,*P*_1_)=*G* *U*(*α*,*P*_1_)+15= max{5.6,7.52}+6.68=14.2.

### **Definition 16**

**(Upper utility of a sequence** ***α*** **in a a time course sequential dataset** ***D*** **)** The upper utility of a sequence *α* in a time course sequential dataset *D* is defined as $\Psi (\alpha,D)=\sum \limits _{P_{r} \in D}\Psi (\alpha, P_{r})$.



Similar to the proof provided in [26], it can be shown that the upper utility of a sequence *α* in a time course sequential dataset *D* is an upper-bound of the true utilities of all the *prefixSUPs* of *α* in *D*. That is, $\forall \beta \gtrsim \alpha, GU(\beta, D) \leq \Psi (\alpha, D)$. The difference here is that in [26], the upper bound is used to prune the search space during the mining process. Here, we argue that this value can be used to select what path should be traced first.

### **Definition 17**

**Priority relationship** Given a time course sequential dataset *D*, a sequence *α* and two genes *G*_*i*_ and *G*_*j*_, regardless of what concatenation process will be used, let $\alpha _{G_{i}}$ be the extended pattern after adding *G*_*i*_ to *α* and let $\alpha _{G_{j}}$ be the pattern after extending *α* using *G*_*j*_. *G*_*i*_ is prior to *G*_*j*_, if and only if $\Psi (\alpha _{G_{i}},D) \geq \Psi (\alpha _{G_{j}},D)$ and it denoted as $G_{i} \lhd G_{j}$.

The priority relationship means that the candidates produced in the same concatenation level may have different rest utility value. Hence, we can sort them in descending order of their rest utility values and the candidates with higher rest utility will be generated before those of having lower rest utility. Therefore, a newly generated candidate may have higher utility and thus, the threshold can be raised sooner. The rationale is that rest utility is an upper bound utilities in of the generated candidates, hence extending the pattern toward the one with higher rest utility may produce patterns with higher utility.

Given a sequence *α* and the *G*_1_,*G*_2_,...,*G*_*n*_ are genes can be added to *α*. We will concatenate genes to *α* according to their priority relationships $G_{p_{1}} \lhd G_{p_{2}} \lhd \dots \lhd G_{p_{n}}$ where $G_{p_{1}}, G_{p_{2}}, \dots, G_{p_{n}}$ is the order to be concatenated.

For example, given *P*_1_ in Table 3 and *α*=〈*G*_3−_〉, we can have one I-Concatenate with *G*_1−_ and its *Ψ* value is 2.08 and three S-Concatenates with *G*_1−_,*G*_2+_ and *G*_3−_ where *Ψ*({*G*_3−_}{*G*_1−_},*P*_1_)=4, *Ψ*({*G*_3−_}{*G*_2+_},*P*_1_)=1.96 and *Ψ*({*G*_3−_}{*G*_3−_},*P*_1_)=0.32. Hence, the priority relationship is: $G^{s}_{1^{-}} \lhd G^{s}_{2^{+}} \lhd G^{i}_{1^{-}} \lhd G^{s}_{3^{-}}$ where *G* ^*s*^ means that *G* is added using S-Concatenate and *G* ^*i*^ means that *G* is added using I-Concatenate process.

The overview of *Top-HUGS* is illustrated in Algorithm 1. Given a time course sequential dataset *D*, *Top-HUGS* builds the *ItemUtilLists* to maintain the information of every gene in each time sample in *D*. Then, it populates *TKList* by applying *PES* strategy based on genes in the *ItemUtilLists* and the sequences in the dataset. Then, *minUtil* is initialized by the utility value of *kth* tuple in *TKList*. Given the initialized *minUtil*, *Top-HUGS* constructs *HUSP-Tree* using *I-Step* and *S-Step*. During the tree construction, whenever a node is going to be extended, we first apply RSO to choose which item should be added first. Once a new node is added to the tree, *TKList* and *minUtil* are updated as explained before. Finally, if the user asks to discover top-k *HUGSs*, *Top-HUGS* returns all the patterns and their *util* values in the *TKList* as top-k *HUGSs* (i.e., *T* *H* *U* *G* *S*_*D*_).

### **Theorem 1**

Given a time course gene sequential database *D*, if a pattern *α* is among the top-k high utility gene regulation sequential patterns, it is returned by Top-HUGS.

### *Proof*

We prove the theorem by showing that the proposed strategies in Top-HUGS never miss a top-k high utility gene regulation sequential pattern.

1. **Baseline procedure**: the only strategy applied in the baseline procedure is raising the threshold during the mining process. We raise the threshold when at least *k* patterns have been inserted to *TKList*. Since the threshold is raised by the utility of *kth* pattern in the list, there are at least *k* patterns in the list. Hence, we do not miss any top-k high utility gene regulation sequential pattern.
2. **PES Strategy**: according to [5], if *α* is a top-k high utility gene regulation sequence, it will not miss by using this strategy.
3. **RSO strategy**: this strategy uses the same approach as above items to raise the threshold. That is, it raises the threshold using the utility of *kth* pattern. The only difference here is in the order of pattern generation. Hence, if a pattern is a top-k high utility pattern it will be generated eventually. Therefore, we do not miss any top-k high utility gene regulation pattern using this strategy.

Since TU-SEQ is an exact method to find all high utility gene regulation patterns [5] and the proposed strategy does not miss any top-k HUGS, if *α* is among the top-k high utility gene regulation sequential patterns, it will be returned by *Top-HUGS*. □

## Results and discussion

In this section, we evaluate the performance of proposed method in finding top-k HUGSs. All the algorithms are implemented in Java. The experiments are conducted on an Intel(R) Core(TM) i7 2.80 GHz computer with 12 GB of RAM. We mine a publicly available dataset *GSE6377* [27] in our experiments. McDunn et al. [27] found 8793 transcriptional changes in 11 ventilator-associated pneumonia patients’ leukocytes across 10 time samples.

### Gene importance for pneumonia

There are several repositories that provide information regarding associations between genes and diseases such as *CTD* [28]. Each repository takes different factors of the phenotype-genotype relationship to investigate gene-disease association and none of them are complete. *DisGeNET* is a platform which aggregates different information resources extracted from the literature to build a comprehensive view of the state of the art knowledge about gene-disease associations. Therefore, we choose the *score* proposed by *DisGeNET* to represent the importance of a gene with respect to a disease. This score includes several variables such as number and type of sources (level of curation, model organisms) and the number of publications supporting the association to rank genes with respect to a specific disease. Table 5 shows top-20 genes w.r.t. *Pneumonia* and their scores.

**Table 5:** Top-20 genes related to pneumonia

Rank	Gene	Rank	Gene	Rank	Gene	Rank	Gene
1	*CAT*	6	*SFTPC*	11	*SFTPA1*	16	*HMGB1*
2	*PDPN*	7	*SFTPB*	12	*CYP2J2*	17	*CR1*
3	*TLR6*	8	*PECAM1*	13	*F2*	18	*MASP2*
4	*TLR2*	9	*ITGB3*	14	*CXCL1*	19	*FCGR2A*
5	*SFTPD*	10	*CXCL2*	15	*MBL2*	20	*IL17A*

The utility of gene *G* in time sample $P_{r}^{d}$ is calculated as follows: $GU\left (G,P_{r}^{d}\right) = GI(G) \times IGU\left (G, P_{r}^{d}\right)$, where *G* *I*(*G*) is the gene importance *G* w.r.t. *Pneumonia* and $IGU\left (G, P_{r}^{d}\right)$ is the internal utility of *G* in time sample *T* *S*_*d*_ in sequence *P*_*r*_. Note that, to calculate utility, any model can be plugged in as desired. The use of more sophisticated the model may further improve the quality of the results.

### The importance of genes with respect to Rheumatoid Arthritis

In addition to *pneumonia*, we also investigate the gene sequential patterns in the same dataset with respect to *Rheumatoid Arthritis*. Table 6 shows top-10 diseases related to *pneumonia*. The second column in this table shows the number of shared genes. Our goal is to show that considering a different disease results different patterns on the same dataset. Therefore, we find gene sequential patterns with respect to *Rheumatoid Arthritis*. Table 7 shows top-20 genes related to *Rheumatoid Arthritis*. Note that this list downloaded from DisGeNET on 2016. It can be seen that there are several genes in common between these diseases.

**Table 6:** Top-10 diseases that share genes with *pneumonia*

Disease name	Shared genes
*Malignant neoplasm of breast*	295
*Breast carcinoma*	285
*Rheumatoid Arthritis*	274
*Asthma*	267
*Carcinogenesis*	258
*Neoplasm metastasis*	258
*Liver carcinoma*	249
*HIV infections*	237

**Table 7:** Top-20 genes related to *Rheumatoid Arthritis*

Rank	Gene	Rank	Gene	Rank	Gene	Rank	Gene
1	*PTPN22*	6	*STAT4*	11	*CCL21*	16	*REL*
2	*TNF*	7	*TRAF1*	12	*IL2RA*	17	*CCR6*
3	*HLA-DRB1*	8	*IRF5*	13	*CDK6*	18	*CIITA*
4	*CTLA4*	9	*SLC22A4*	14	*NFKBIL1*	19	*MMEL1*
5	*PADI4*	10	*CD40*	15	*KIF5A*	20	*CD244*

The utility of gene *G* in time sample $P_{r}^{d}$ is calculated as follows: $GU\left (G,P_{r}^{d}\right) = GI(G) \times IGU\left (G, P_{r}^{d}\right)$, where *G* *I*(*G*) is the importance of *G* w.r.t. *Rheumatoid Arthritis* retrieved from *DisGeNET* and $IGU\left (G, P_{r}^{d}\right)$ is the expression value of *G* in time sample *T* *S*_*d*_ in sequence *P*_*r*_.

### The importance of genes with respect to Asthma

We also consider the gene sequential patterns in the same dataset with respect to *Asthma*. Table 8 shows top-20 genes related to *Asthma*. It can be seen that there are several genes in common between these diseases. Similar to the previous subsection, we calculate the utility of gene *G* in time sample $P_{r}^{d}$ as follows: $GU\left (G,P_{r}^{d}\right) = GI(G) \times IGU\left (G, P_{r}^{d}\right)$, where *G* *I*(*G*) is the importance of *G* w.r.t. *Asthma* retrieved from *DisGeNET* and $IGU\left (G, P_{r}^{d}\right)$ is the expression value of *G* in time sample *T* *S*_*d*_ in sequence *P*_*r*_.

**Table 8:** Top-20 genes related to *Asthma*

Rank	Gene	Rank	Gene	Rank	Gene	Rank	Gene
1	*TBCE*	6	*CXCL8*	11	*TGFB1*	16	*IFNG*
2	*SDC4*	7	*IRAK4*	12	*CFTR*	17	*IL10*
3	*MBL2*	8	*IL6*	13	*NOD2*	18	*PTGS2*
4	*TLR2*	9	*MYD88*	14	*TNF*	19	*CAMP*
5	*TLR4*	10	*CALCA*	15	*CRP*	20	*ABL1*

### Pneumonia: Top-k HUGSs versus Top-k FGSs

In this section, we investigate if patterns discovered by *TU-SEQ* and *Top-HUGS* contain potential genes/regulations which have not been reported by existing methods. The algorithms are ran to extract top-k *HUGSs* with respect to *Pneumonia*. Moreover, a recent method called *CTGR-Span* [2] is ran to find frequent gene regulation sequential patterns (i.e., *FGSs*) from the dataset. Given a discovered pattern *α* and a disease *dis*, the quality of the results is evaluated by *popularity of a sequence* [29] which is defined $Pop(\alpha, dis) = \frac {\sum \limits _{i \in \alpha } w(i, dis)}{|\alpha |}$, where *w*(*i*,*d* *i* *s*) is the importance of the popular gene *i* for disease *dis*. Without loss of generality, the genes presented in Table 5 are considered as popular genes and *w*(*i*,*d* *i* *s*)=20−*r* *a* *n* *k*(*i*,*d* *i* *s*)+1. For the genes which are not presented in the list, *w*(*i*,*d* *i* *s*)=1.

Table 9 shows top-4 *HUGSs* extracted by *TU-SEQ* and top-4 *FGSs* extracted by *CTGR-Span*, sorted by the utility and support respectively. Since the output patterns for both *TU-SEQ* and *Top-HUGS* are the same, we only present the results of *Top-HUGS*. Table 9 confirms that the frequent sequences are not necessarily significant w.r.t. the disease even though their support value is high. This is due to the fact that such patterns are identified based on their frequency which is not informative enough. Moreover, patterns returned by *Top-HUGS* are relatively significant. Such patterns help biologists choose relevant sequences with respect to a specific disease and also identify the relationships between important genes and the unknown genes.

**Table 9:** Top-4 HUGSs versus Top-4 FGSs with respect to *Pneumonia*

Algorithm	ID.	Sequence of genes (e.g., *α*)	*Support*	*Util*
Top-HUGS	*H* *U* *G* *S*_1_	(CAT) (CAT MBL2) (CAT)	9	250600
	*H* *U* *G* *S*_2_	(GOLPH3 PDPN) (CAT) (PDPN) (CAT) (PDPN)	9	250325
	*H* *U* *G* *S*_3_	(CAT MBL2) (CAT) (CAT)	9	249741
	*H* *U* *G* *S*_4_	(PDPN)(CAT) (PDPN) (CAT) (PDPN)	9	243037
CTGR-Span	*F* *G* *S*_1_	(LCN2 S100A12) (LCDN2)	11	59981
	*F* *G* *S*_2_	(LCN2) (S100A12) (CSF3 LCN2)	11	59962
	*F* *G* *S*_3_	(CSF3 S100A12)	11	59931
	*F* *G* *S*_4_	(LCN2 S100A12) (LCN2 S100A12)	11	58514

### Rheumatoid Arthritis: Top-k HUGSs comparison with Top-k FGSs

Table 10 shows top-4 *HUGSs* and top-4 *FGSs* with respect to *Rheumatoid Arthritis*, sorted by the utility and support respectively. This table asserts that the frequent sequences are not necessarily popular w.r.t. *Rheumatoid Arthritis*. In this figure, *TU-SEQ* finds the patterns whose popularity is high compared to those of returned by CTGR-Span.

**Table 10:** Top-4 HUGSs versus Top-4 FGSs with respect to *Rheumatoid Arthritis*

Algorithm	ID.	Sequence of genes (e.g., *α*)	*Support*	*Util*
Top-HUGS	*H* *U* *G* *S*_1_	(TRAF1 CTLA4 IL1B) (IL2RA CD40) (TRAF1 PADI4 CTLA4) (STAT4 IL2RA)	6	5857
	*H* *U* *G* *S*_2_	(TRAF1 CTLA4 IL1B) (PTPN2 CD40) (TRAF1 PADI4 CTLA4) (STAT4 IL2RA)	6	5856
	*H* *U* *G* *S*_3_	(TRAF1 CTLA4 IL1B) (CD40) (TRAF1 PADI4 CTLA4 IL1B) (STAT4 IL2RA)	6	5843
	*H* *U* *G* *S*_4_	(TRAF1 CTLA4 IL1B) (IL2RA PTPN2 CD40) (TRAF1 PADI4 CTLA4) (STAT4)	6	5834
CTGR-Span	*F* *G* *S*_1_	(CTLA4) (IL1B)	11	2981
	*F* *G* *S*_2_	(PTPN2 PADI4) (TRAF1) (PTPN2)	11	2964
	*F* *G* *S*_3_	(TRAF1) (TRAF1) (PTPN2)	11	2961
	*F* *G* *S*_4_	(ANXA3) (PTPN2 PADI4)	11	2947

### Asthma: Top-k HUGSs comparison with Top-k FGSs

Table 11 shows top-4 *HUGSs* and top-4 *FGSs* with respect to *Asthma*. According to this figure, even though top-4 HUGS (the average support is 9) are not as frequent as top-4 FGS (the average support is 11), their utility value is 4 times higher in average. Since the utility is defined with respect to the disease, we can claim that the results obtained by TU-SEQ are more important than those of obtained by *CTGR-Span*.

**Table 11:** Top-4 HUGSs versus Top-4 FGSs with respect to Asthma

Algorithm	ID.	Sequence of genes (e.g., *α*)	*Support*	*Util*
Top-HUGS	*H* *U* *G* *S*_1_	(TAF9 KCMF1) (TBCE) (TAF9) (TAF9)	10	218301
	*H* *U* *G* *S*_2_	(TAF9) (TBCE) (TAF9) (VAMP4)	10	215541
	*H* *U* *G* *S*_3_	(TAF9) (TBCE) (TAF9) (TBCE)	10	207292
	*H* *U* *G* *S*_4_	(TAF9) (TBCE) (TBCE)	10	201802
CTGR-Span	*F* *G* *S*_1_	(CAMP) (CAMP) (CAMP)	11	39216
	*F* *G* *S*_2_	(CAMP)	11	86800
	*F* *G* *S*_3_	(CAMP) (CAMP) (CAMP) (CAMP)	11	55486
	*F* *G* *S*_4_	(CAMP) (CAMP) (FCN2 CAMP) (CAMP)	11	15136

### Quantitative evaluation

Given top-1000 patterns found by the methods, the average value of utility (i.e., *GU*), *Pop* and *Sup* are calculated and presented in Table 12. The last two columns show harmonic mean of *(GU, Pop)* and *(Sup, Pop)*. They are computed as follows: $GU-Pop = 2 \times \frac {GU \times Pop}{GU + Pop}$, $Sup-Pop = 2 \times \frac {Sup \times Pop}{Sup + Pop}$. Top-HUGS achieved higher values of these measures since they are not only much more relevant to the disease, but also they are frequent enough.

**Table 12:** The average value of *Sup*, *GU*, *Pop*, *GU-Pop* and *Sup-Pop* for top-1000 sequences returned by the method

Method	Sup	GU	Pop	GU-Pop	Sup-Pop
Top-HUGS	5	198939	12.5	24.96	7.32
CTGR-Span	10	44691	1.02	2.05	1.86

### Efficiency of TU-SEQ and Top-HUGS

In this section, the performance of the algorithms are evaluated in terms of (1) *Run Time (sec.)*: the total execution time of the algorithms, and (2) *Memory Usage (MB)*: the average memory consumption per window.

Since our methods are the only methods for mining *top-k utility-based gene regulation sequential patterns*, we implemented three different versions of *Top-HUGS*.

Table 13 presents these versions. The first method is the baseline procedure (i.e., *Top-HUGS *_Base_) which does not use any of the proposed raising strategies to set the threshold. TU-SEQ the proposed method in [5] is an extended version of *TU-SEQ *_Base_ that applies *PES* strategy to initialize the threshold. Top-HUGS _*R*_ uses *RSO* to raise the threshold but does not apply PES strategy. We also use the threshold-based approach (i.e., *HUSP-Miner*) proposed in [30] as another baseline approach.

**Table 13:** Different versions of Top-HUGS

Method	Baseline	PES	RSO
*T* *o* *p*−*H* *U* *G* *S*_*Base*_	✓	×	×
*TU-SEQ*	✓	✓	×
*T* *o* *p*−*H* *U* *G* *S*_*R*_	✓	×	✓
*T* *o* *p*−*H* *U* *G* *S*	✓	✓	✓

After getting the *utility* of the k-th pattern which is the optimal minimum threshold in Definition 9, we use this value as the threshold to run *HUSP-Miner*.

**Fig. 3:**
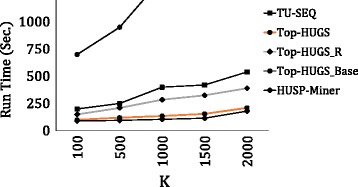
Run time on the GSE3677 Dataset

We compare *Top-HUGS* with Top-HUGS _*Base*_ and *HUSP-Miner* on the *GSE6377* dataset. The results in terms of running time are presented in Fig. 3. The results confirm that *Top-HUGS* is significantly (more than 5 times) faster than *Top-HUGS *_Base_. For larger values of *k*, *Top-HUGS *_Base_ cannot even return the patterns within 12+ hours. Moreover, the time performance gap between *Top-HUGS* and *Top-HUGS *_Base_ increases with larger values of *k*. The results show that *PES* and *RSO* strategies are effective for mining top-k HUGSs.

**Fig. 4:**
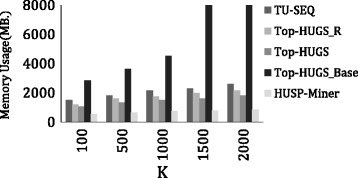
Memory usage on the GSE3677 Dataset

We also evaluate the performance of the algorithms in terms of memory usage. The results are presented in Fig. 4. *Top-HUGS* consumes less memory than *Top-HUGS *_Base_, *TU-SEQ* and *Top-HUGS *_R_. This is due to the fact that *Top-HUGS* produces a smaller search space than the other versions since it applies both strategies and can raise the threshold quicker than the other methods. Since the tree construction is the same for all the methods, the main factor in memory consumption is the threshold used by the method during the tree construction. *HUSP-Miner* consumes less memory since it uses the optimal threshold (i.e., *m* *i* *n* *U* *t* *i* *l*_*opt*_), thus prunes prunes the search space efficiently.

### Demonstration

A web interface of our system is developed in Java (Please go to http://mzk.eecs.yorku.ca:8080/GeneAssociation/). This is the first web interface to mine *top-k high utility gene regulation sequential patterns*. The system uses *GSE6377* dataset as the input dataset to find top-k *HUGSs*. According to *DisGNET*, *Athma* and *Rheumatoid Arthritis* are among top 10 diseases that share genes with *Pneumonia*. Therefore, we also provide top-k HUGSs with respect to *Athma* and *Rheumatoid Arthritis*. Moreover, the patterns discovered by *CTGR-Span* [2] are compared. In the first page of the interface, the user can specify *disease*, *ranking measure*, number of output gene regulation sequences (i.e., *k*) and *discovery method(s)*.

In the demonstration, users can compare the algorithms in the following aspects:

1. *Useful results:* Our method based on the utility model produces more meaningful sequences than the other method.
2. *Top-k HUGS:* The sequences retrieved by the methods are provided in a meaningful graphical presentation.
3. *Additional information:* Additional information such as values for the other measures than the selected one for ranking and top-20 genes related to the selected disease.

**Fig. 5:**
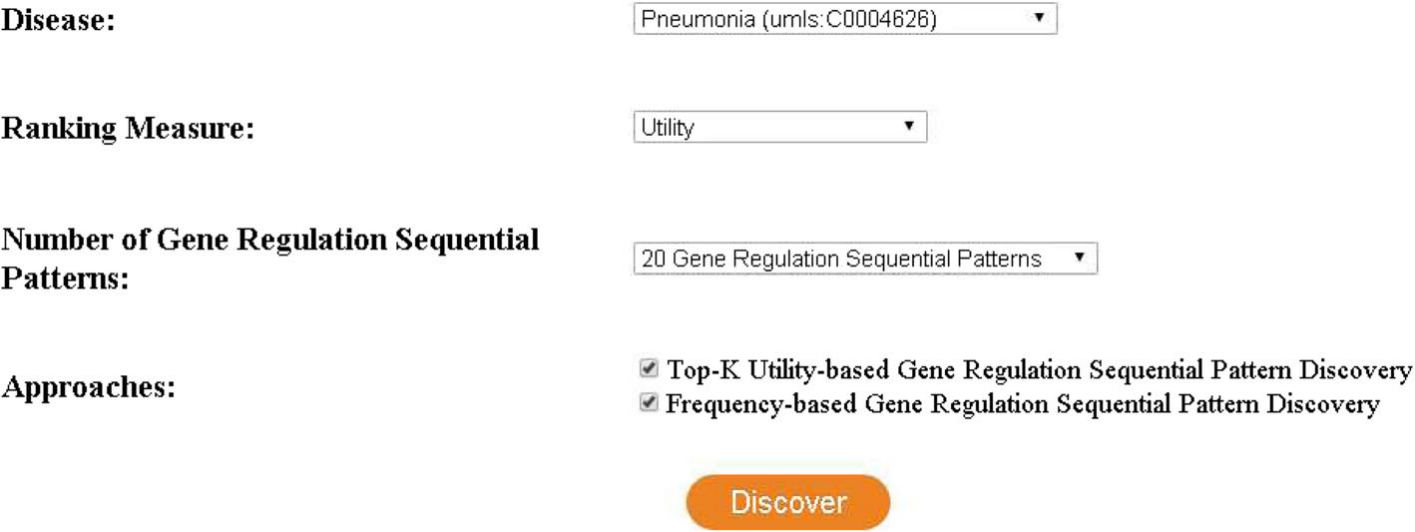
First page of the system with parameters

**Fig. 6:**
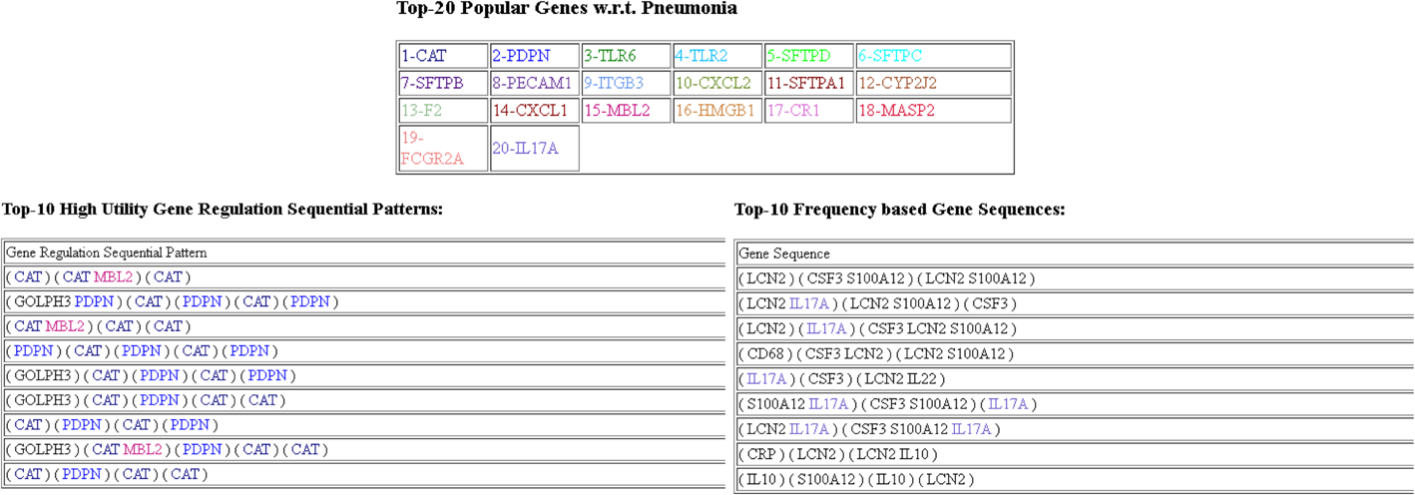
Second page of the system for discovered patterns

Figures 5 and 6 show the first and second page of the system. In the first page, the user selects the disease, the ranking method and the number of output patterns. The second page visualizes the results obtained from the dataset based on the given parameters. The genes in each pattern are colored according to top-20 genes with respect to the selected disease.

## Conclusion

In this paper, we defined the problem of *top-k utility-based gene regulation sequential pattern discovery* to find patterns with stronger meanings in biology. By solving this problem, we addressed the limitations of previous frequency-based gene regulation sequential pattern mining methods. We first proposed a *utility* model by considering the importance of genes with respect to a disease and their temporal behaviour. Then, using the utility model, we proposed two efficient algorithms called *TU-SEQ* and *Top-HUGS* to find top-k high utility gene regulation sequential patterns. To the best of our knowledge, existing methods for mining gene regulation sequential patterns are threshold-based methods and assume all genes have similar importance, which is often not true in real life scenarios. Our experiments suggest that *TU-SEQ* and *Top-HUGS* are much more efficient and scalable than baseline algorithms for top-k high utility gene sequential pattern discovery. We also showed that *Top-HUGS* is an effective tools to provide biologists with further insights into the relationships of gene regulatory events and interactions in biological studies with respect to a specific disease.

## References

[1] Hirschhorn JN, Daly MJ (2005). Genome-wide association studies for common diseases and complex traits. Nat Rev Genet.

[2] Cheng CP, Liu YC, Tsai YL, Tseng VS (2013). An efficient method for mining cross-timepoint gene regulation sequential patterns from time course gene expression datasets. BMC Bioinformatics.

[3] Bringay S, Roche M, Teisseire M, Poncelet P, Rassoul RA, Verdier JM, Devau G (2010). Discovering novelty in sequential patterns: application for analysis of microarray data on alzheimer disease. MedInfo: Congress on Medical Informatics.

[4] Georgii E, Richter L, Rückert U, Kramer S (2005). Analyzing microarray data using quantitative association rules. Bioinformatics.

[5] Zihayat M, Davoudi H, An A. Top-k utility-based gene regulation sequential pattern discovery. In: 2016 IEEE International Conference on Bioinformatics and Biomedicine (BIBM).2016. p. 266–73. doi:10.1109/BIBM.2016.7822529.

[6] Ayres J, Flannick J, Gehrke J, Yiu T (2002). Sequential pattern mining using a bitmap representation. Proc. of ACM SIGKDD Intl. Conf. on Knowledge Discovery and Data Mining..

[7] Agrawal R, Srikant R. Mining sequential patterns. In: Proceedings of the Eleventh International Conference on Data Engineering. ACM;: 1995. p. 3–14. doi:10.1109/ICDE.1995.380415.

[8] Han J, Pei J, Mortazavi-Asl B, Chen Q, Dayal U, Hsu M (2010). Freespan: Frequent pattern-projected sequential pattern mining. In Proc.of ACM SIGKDD International Conference on Knowledge Discovery and Data Mining.

[9] Pei J, Han J, Mortazavi-Asl B, Chen Q, Dayal U, Hsu M (2004). Mining sequential patterns by pattern-growth: The prefixspan approach. TKDE.

[10] Srikant R, Agrawal R, Apers P, Bouzeghoub M, Gardarin G (1996). Mining sequential patterns: Generalizations and performance improvements.. Advances in Database Technology — EDBT ’96: 5th International Conference on Extending Database Technology Avignon, Proceedings.

[11] Zaki MJ (2001). SPADE: An efficient algorithm for mining frequent sequences. Mach Learn.

[12] Gupta JM, Han J, Kumar P, Krishna PR, Raju SD (2012). Applications of pattern discovery using sequential data mining.. Pattern Discovery Using Sequence Data Mining: Applications and Studies.

[13] Ahmed CF, Tanbeer SK, Jeong B (2010). A novel approach for mining high-utility sequential patterns in sequence databases. ETRI J.

[14] Ahmed CF, Tanbeer SK, Jeong B (2011). A framework for mining high utility web access sequences. IETE J.

[15] Shie B, Hsiao H-F, Tseng VS (2013). Efficient algorithms for discovering high utility user behavior patterns in mobile commerce environments. Knowl Inf Syst.

[16] Yin J, Zheng Z, Cao L (2012). Uspan: An efficient algorithm for mining high utility sequential patterns. Proceedings of the 18th ACM SIGKDD International Conference on Knowledge Discovery and Data Mining.

[17] Zihayat M, Wu C-W, An A, Tseng VS (2015). Mining high utility sequential patterns from evolving data streams. Proceedings of the ASE BigData & SocialInformatics 2015.

[18] Liu Y, Liao W-k, Choudhary A (2005). A fast high utility itemsets mining algorithm. Proceedings of the 1st International Workshop on Utility-based Data Mining.

[19] Ahmed CF, Tanbeer SK, Jeong BS (2012). Interactive mining of high utility patterns over data streams. Expert Syst Appl.

[20] Tseng VS, Shie BE, Wu CW, Yu PS (2013). Efficient algorithms for mining high utility itemsets from transactional databases. IEEE Trans Knowl Data Eng.

[21] Liu M, Qu J (2012). Mining high utility itemsets without candidate generation. Proceedings of the 21st ACM International Conference on Information and Knowledge Management..

[22] Fournier-Viger P, Wu C-W, Zida S, Tseng VS, Andreasen T, Christiansen H, Cubero J-C, Ra ZW (2014). FHM: Faster high-utility itemset mining using estimated utility co-occurrence pruning.. Foundations of Intelligent Systems: 21st International Symposium, ISMIS 2014, Proceedings.

[23] Creighton C, Hanash S (2003). Mining gene expression databases for association rules. Bioinformatics.

[24] Chen Q, Chen Y-PP (2006). Mining frequent patterns for amp-activated protein kinase regulation on skeletal muscle. BMC Bioinformatics.

[25] Hsu C-M, Chen C-Y, Hsu C-C, Liu B-J, Ng W-K, Kitsuregawa M, Li Jianzhong, Chang K (2006). Efficient discovery of structural motifs from protein sequences with combination of flexible intra-and inter-block gap constraints.. Pacific-Asia Conference on Knowledge Discovery and Data Mining.

[26] Zihayat M, Chen Y, An A (2017). Memory-adaptive high utility sequential pattern mining over data streams. Mach Learn.

[27] McDunn J, Husain K, Polpitiya A, Burykin A, Li Q, Schierding W, Lin N, Dixon D, Zhang W (2008). Plasticity of the systemic inflammatory response to acute infection during critical illness: development of the riboleukogram. Plos ONE.

[28] Davis AP, Murphy CG, Johnson R, Lay JM, Lennon-Hopkins K, Saraceni-Richards C, Sciaky D, King BL, Rosenstein MC, Wiegers TC (2013). The comparative toxicogenomics database: update 2013. Nucleic Acids Res.

[29] Bringay S, Roche M, Teisseire M, Poncelet P, Abdel Rassoul R, Verdier JM, Devau G (2010). Discovering novelty in sequential patterns: application for analysis of microarray data on alzheimer disease. Stud Health Technol Inform..

[30] Zihayat M, Wu CW, An A, Tseng VS, Lin C (2017). Efficiently mining high utility sequential patterns in static and streaming data. Intel Data Anal.

